# Cardiometabolic Biomarkers for Disease Severity Prediction in Metabolic Dysfunction‐Associated Steatotic Liver Disease

**DOI:** 10.1002/kjm2.70261

**Published:** 2026-07-13

**Authors:** Jee‐Fu Huang, Chi‐Yi Chen, Ming‐Jong Bair, Tzu‐Chun Lin, Ming‐Lun Yeh, Pei‐Chien Tsai, Yu‐Ju Wei, Chih‐Wen Wang, Po‐Chen Liang, Ming‐Yen Hsieh, Mei‐Hsuan Lee, Chia‐Yen Dai, Chung‐Feng Huang, Ming‐Lung Yu, Wan‐Long Chuang

**Affiliations:** ^1^ Hepatobiliary Division, Department of Internal Medicine, Kaohsiung Medical University Hospital Kaohsiung Medical University Kaohsiung Taiwan; ^2^ College of Medicine Kaohsiung Medical University Kaohsiung Taiwan; ^3^ Center for Metabolic Disorders and Obesity Kaohsiung Medical University Kaohsiung Taiwan; ^4^ Division of Gastroenterology and Hepatology, Department of Medicine Ditmanson Medical Foundation Chiayi Christian Hospital Chiayi Taiwan; ^5^ Division of Gastroenterology, Department of Internal Medicine Taitung Mackay Memorial Hospital Taitung Taiwan; ^6^ Mackay Medical College New Taipei City Taiwan; ^7^ Doctoral Program of Clinical and Experimental Medicine, College of Medicine and Center of Excellence for Metabolic Associated Fatty Liver Disease National Sun Yat‐sen University Kaohsiung Taiwan; ^8^ Institute of Clinical Medicine National Yang Ming Chiao Tung University Taipei Taiwan

**Keywords:** biomarkers, cardiometabolic risks, liver fibrosis, metabolic dysfunction‐associated steatotic liver disease, type 2 diabetes mellitus

## Abstract

The impacts of cardiometabolic risk factors (CMRFs) and the biomarkers on disease severity in metabolic dysfunction‐associated steatotic liver disease (MASLD) remain elusive. Accordingly, a multicenter study by recruiting MASLD patients from a nationwide registry database was conducted with a total of 2882 MASLD patients (54.1% males, mean age = 55.6 ± 13.9 years, mean body mass index [BMI] = 28.1 ± 4.9 kg/m^2^) being recruited. The proportions of patients carrying CMRFs of overweight/obesity, hypertension, hypertriglyceridemia, low high‐density lipoprotein cholesterol level, and diabetes/prediabetes were 91.2%, 74.5%, 54.8%, 59.6%, and 74.5%, respectively. There was a significant linear trend between significant fibrosis and CMRFs (5.1%, 27%, and 54.6% in patients with one CMRF, 3 CMRFs, and ≥ 4 CMRFs, respectively, *p* = 0.009). The adjusted odds ratios (aOR) with 95% confidence intervals (CIs) for advanced fibrosis in patients of hemoglobin A1c (HbA1c) 5.7%–6.5%, 6.5%–8.0%, and > 8.0% were 1.02 (95% CI = 0.68–1.53, *p* = 0.93), 1.83 (95% CI = 1.16–2.88, *p* = 0.01), and 2.78 (95% CI = 1.45–5.34, *p* = 0.002), respectively. The significant risk factors for advanced fibrosis in MASLD were age > 60 years (aOR = 12.08, 95% CI = 6.23–23.46, *p* < 0.001), elevated transaminase level (aOR = 1.06, 95% CI = 1.05–1.09, *p* < 0.001), and diabetes (aOR = 1.51, 95% CI = 1.02–2.26, *p* = 0.04). In conclusion, there was a significant linear association between advanced fibrosis and the items of CMRFs. The HbA1c level could serve as a predictive biomarker for advanced fibrosis in MASLD with the implication that diabetes control is mandatory for patients with MASLD.

**Abbreviations**
aOR: adjusted odds ratios
BMI: body mass index
CI: confidence interval
CVD: cardiovascular diseases
FIB‐4: fibrosis‐4 index
FLI: fatty liver index
FPG: fasting plasma glucose
HbA1c: hemoglobin A1c
HCC: hepatocellular carcinoma
HDL‐C: high‐density lipoprotein cholesterol
hs‐CRP: high‐sensitivity C‐reactive protein
IR: insulin resistance
MASLD: metabolic dysfunction‐associated steatotic liver disease
SLD: steatotic liver disease
T2DM: type 2 diabetes mellitus

## 1 Introduction

Metabolic dysfunction‐associated steatotic liver disease (MASLD) represents a spectrum of liver disorders ranging from simple steatosis to steatohepatitis and advanced fibrosis [1]. MASLD is closely associated with metabolic disorders such as type 2 diabetes mellitus (T2DM), dyslipidemia, obesity, and hypertension [2, 3], all of which predispose MASLD patients to hepatocyte injuries, fibrosis, cirrhosis, and potential development of hepatocellular carcinoma (HCC). MASLD‐linked metabolic disorders and obesity are strongly associated with both insulin resistance (IR) and T2DM that commonly lead to chronic macrovascular and microvascular complications [4, 5]. Cardiovascular disease (CVD) is one of the complications contributing to the major causes of death in MASLD [6, 7]; therefore, the investigation of the features and cardiometabolic risk factors (CMRFs) in MASLD is essential for adequate and appropriate patient management.

Additionally, the precise pathophysiology, clinical progression, and long‐term outcomes are still not well elucidated, with the presentation and associated comorbidities varying across ethnicities and regions in MASLD patients. For example, the epidemic of MASLD continues to rapidly progress in recent decades in the Asia‐Pacific area paralleling the rapid Westernization of the region. However, the relatively lower body mass index (BMI) in Asians is not protective from metabolic alterations, while MASLD, T2DM, and metabolic disorders are more prevalent in Asians than other races with the same BMI value [8, 9]. As a result, the exploration of reliable risk prediction tools for disease severity is a must for elucidating the pathogenic mechanisms on a regional basis where such clarifications will pave the way for both diagnosis and risk stratification.

Some direct or indirect inflammatory biomarkers have a prognostic value for the development of CVD independent of conventional risk factors and might be useful for identifying patients at high risk of future CVD who could benefit from specific treatment to reduce this risk [10]. Nevertheless, the predictive power of the common biomarkers for disease severity of MASLD such as the liver fibrosis stage has rarely been investigated, and such elucidation would provide insightful mutual translation clues for translational research, which in turn could speed the elucidation of the pathogenic mechanisms of the complex metabolic disorder. Additionally, such efforts could provide useful information for precision patient management.

Consequently, the study aimed to evaluate the interplay between MASLD and CMRFs while also exploring the potential cardiometabolic risk biomarkers for advanced fibrosis prediction in MASLD patients.

## 2 Materials and Methods

### 2.1 Study Design and Participants

This was a multicenter, retrospective cohort study conducted using a nationwide integrated registry platform database of patients with SLD in Taiwan between Sep 2022 and Feb 2025. The primary goal of the platform was to establish a comprehensive clinical database that will track the progression, biochemical markers, and long‐term outcomes of MASLD patients. All adult patients with MASLD were eligible for inclusion, with those meeting the following criteria being excluded: (1) patients with a prior diagnosis of HCC within 5 years upon the diagnosis of SLD; (2) patients with other causes of malignancy except basal cell carcinoma of skin; (3) decompensated liver states; (4) any evidence of organ failure; (5) patients with other secondary causes potentially leading to steatosis in the liver (drugs, autoimmune liver diseases, etc.); or (6) patients with a history of alcohol consumption exceeding 20 g/day for females and 30 g/day for males. Baseline data collected included demographic characteristics, clinical variables, laboratory values, and liver fibrosis as assessed by the fibrosis index (FIB)‐4 score.

### 2.2 MASLD Definition

MASLD was defined by the diagnostic criteria according to the current guidelines [1, 11]. Briefly, the MASLD patients must have SLD in addition to the presence of at least one of the five CMRFs, including (1) BMI ≥ 23 kg/m^2^; (2) fasting plasma glucose (FPG) ≥ 100 mg/dL, glycated hemoglobin (HbA1c) ≥ 5.7% or T2DM history with or without treatment; (3) blood pressure ≥ 130/85 mmHg or antihypertensive drug treatment; (4) plasma triglycerides ≥ 150 mg/dL [12] or lipid‐lowering treatment; and (5) plasma high‐density lipoprotein cholesterol (HDL‐C) ≤ 40 mg/dL for males and ≤ 50 mg/dL for females or lipid‐lowering treatment. The formula for the HOMA‐IR was FPG (mg/dL) × fasting insulin level (μU/mL)/405.

### 2.3 SLD and Fibrosis Assessment

Abdominal sonography was performed for each participant by well‐experienced and licensed hepatologists at the same institution to ensure interobserver consistency. The precise recruitment of patients with SLD was further validated by the fatty liver index (FLI) for the undetermined patients by sonography [13] The blood fibrosis test FIB‐4 was calculated according to the following formula: [age × AST (IU/L)]/[platelets (10^9^/L) × ALT (IU/L)^1/2^] [14]. A cutoff of > 2.67 was defined as advanced fibrosis stage (F3‐4). Non‐advanced fibrosis was classified as mild (FIB‐4 ≤ 1.3) and intermediate (1.3 < FIB‐4 ≤ 2.67).

### 2.4 Statistical Analysis

Continuous variables were expressed as means ± standard deviations and compared using Student *t*‐tests or Mann–Whitney *U* tests, depending on data distribution. Categorical variables were presented as frequencies and percentages, with comparisons performed using chi‐square tests, while descriptive statistics were used to depict the baseline characteristics of the study cohort. Multivariate logistic regression was performed to identify independent risk factors for advanced fibrosis, adjusting for potential confounders. The results were reported as adjusted odds ratios (aOR) with 95% confidence intervals (CIs). Statistical significance was set at *p* < 0.05. Analyses were performed using SPSS software (Version 26, IBM Corp.).

## 3 Results

### 3.1 Baseline Characteristics

**FIGURE 1:**
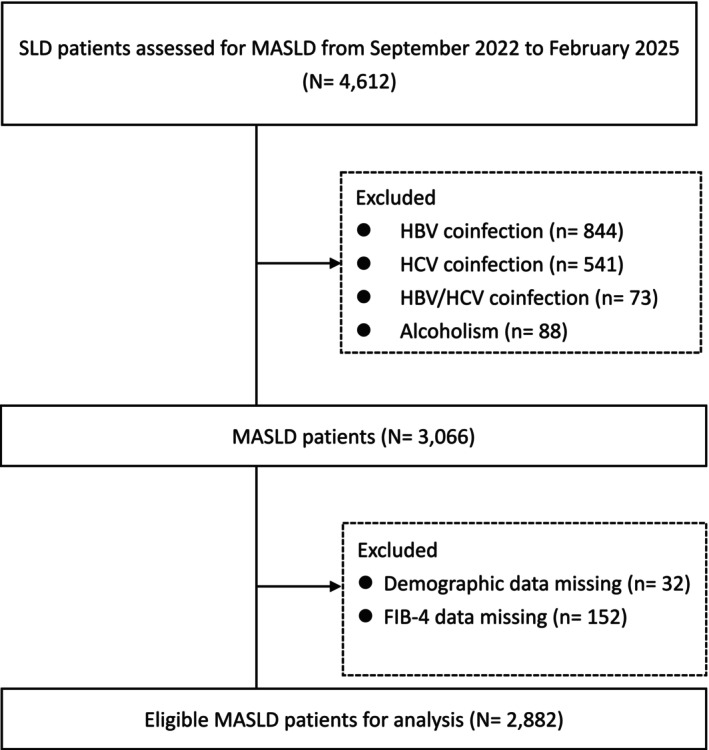
Flowchart of the study.

A total of 4612 SLD patients were recruited for assessment. Excluding those who had viral hepatitis infections, excessive alcohol use, or data missing, a total of 2882 MASLD patients were included in the study (54.1% males, mean age = 55.6 ± 13.9 years) (Figure 1). Their mean BMI was 28.1 ± 4.9 kg/m^2^, and 89% of patients had BMI ≥ 23 kg/m^2^. A total of 2274 (78.9%) had elevated ALT levels. T2DM was diagnosed in 913 (31.7%) patients, and 95.6% of them received anti‐diabetes drugs. High IR (HOMA‐IR > 2.5) was observed in 48.6% of patients. The proportions of patients carrying CMRFs of overweight/obesity, hypertension, hyper‐TG, low HDL‐C, and glucose abnormalities were 91.2%, 74.5%, 54.8%, 59.6%, and 74.5%, respectively (Table 1).

**TABLE 1:** Characteristics of the MASLD patients (*N* = 2882).

Variables	Total
Age (years)	55.6 ± 13.9
≥ 60	1284 (44.6)
Male	1558 (54.1)
BMI (kg/m^2^)	28.1 ± 4.9
≥ 23	2566 (89.0)
Waist circumference (cm)	93.5 ± 11.7
> 90 (male) or > 80 (female)	2132 (75.0)
SBP (mmHg)	133.8 ± 16.7
DBP (mmHg)	80.5 ± 12.1
SBP ≥ 130 or DBP ≥ 85	1826 (64.1)
Smoking	299 (10.4)
Alcohol use	177 (6.1)
Diabetes	913 (31.7)
Anti‐diabetes drug use	703 (95.6)
Hypertension	1193 (41.4)
Drug use	1081 (90.6)
Hyperlipidemia	1190 (41.3)
Drug use	981 (82.4)
AST (IU/L)	32.8 ± 25.4
ALT (IU/L)	45.2 ± 44.4
>ULN	2274 (78.9)
Platelet count (×10^3^/μL)	249.6 ± 66.7
Triglycerides (mg/dL)	144.3 ± 90.1
Total cholesterol (mg/dL)	184.6 ± 38.5
HDL‐C (mg/dL)	50.0 ± 15.6
LDL‐C (mg/dL)	113.3 ± 35.3
Hs‐CRP	1.5 ± 6.7
FPG (mg/dL)	107.0 ± 25.0
HbA1c (%)	6.1 ± 0.9
Insulin (μU/mL)	12.6 ± 13.6
HOMA‐IR	3.5 ± 4.3
≥ 2.5	1273 (48.6)
Creatinine (mg/dL)	0.88 ± 0.52
eGFR (mL/min/1.73 m^2^)	93.9 ± 25.0
< 60	178 (6.3)
FIB‐4	1.31 ± 1.24
> 1.3	1073 (37.2)
> 2.67	152 (5.3)
CMRFs	
Overweight/obesity	2627 (91.2)
Hypertension	2145 (74.5)
Hyper‐TG	1580 (54.8)
Low HDL‐C	1717 (59.6)
Glucose abnormalities	2146 (74.5)

*Note:* Parenthesis indicates percentage.

Abbreviations: ALT, alanine aminotransferase; AST, aspartate aminotransferase; BMI, body mass index; CMRFs, cardiometabolic risk factors; DBP, diastolic blood pressure; eGFR, estimated glomerular filtration rate; FIB‐4, fibrosis‐4 index; FPG, fasting plasma glucose; HbA1c, glycated hemoglobin; HDL‐C, high‐density lipoprotein cholesterol; HOMA‐IR, homeostatic model assessment for insulin resistance; Hs‐CRP, high‐sensitivity C‐reactive protein; LDL‐C, low‐density lipoprotein cholesterol; MASLD, metabolic dysfunction‐associated steatotic liver disease; SBP, systolic blood pressure; ULN, upper limit of normal.

### 3.2 T2DM vs. Non‐T2DM Patients

There were 913 (31.7%) T2DM patients and 1969 non‐T2DM patients (Table 2). T2DM patients were significantly older (mean age: 60.0 ± 12.4 years, *p* < 0.001) and had a higher BMI (28.8 ± 5.1 kg/m^2^ vs. non‐T2DM: 27.7 ± 4.8 kg/m^2^, *p* < 0.001) than the non‐T2DM counterparts. The T2DM group had a higher prevalence of hypertension (59.2% vs. 33.2%, *p* < 0.001) and dyslipidemia (55.9% vs. 34.6%, *p* < 0.001) than the non‐T2DM group. They also had a lower mean eGFR (90.3 ± 28.9 vs. 95.5 ± 22.8 mL/min/1.73m^2^, *p* < 0.001) and a higher proportion of chronic renal disease stages, defined as eGFR < 60 mL/min/1.73 m^2^ (10.9% vs. 4.1%, *p* < 0.001).

**TABLE 2:** Characteristics of the MASLD patients stratified by diabetes.

Variables	T2DM (+) (*n* = 913, 31.7%)	T2DM (−) (*n* = 1969, 68.3%)	*p*
Age (years)	60.0 ± 12.4	53.6 ± 14.1	< 0.001
≥ 60	540 (59.2)	744 (37.8)	< 0.001
Male	512 (56.1)	1046 (53.1)	0.138
BMI (kg/m^2^)	28.8 ± 5.1	27.7 ± 4.8	< 0.001
≥ 23	831 (91.0)	1735 (88.1)	0.018
Waist circumference (cm)	96.1 ± 12.0	92.2 ± 11.4	< 0.001
> 90 (M) or > 80 (F)	737 (81.9)	1395 (71.9)	< 0.001
Smoking	99 (10.8)	200 (10.2)	0.579
Hypertension	540 (59.2)	653 (33.2)	< 0.001
Hyperlipidemia	510 (55.9)	680 (34.6)	< 0.001
AST (IU/L)	34.2 ± 22.4	32.1 ± 26.6	0.030
ALT (IU/L)	45.6 ± 39.5	45.0 ± 46.6	0.736
Platelet count (×10^3^/μL)	241.8 ± 68.2	253.2 ± 65.7	< 0.001
Triglycerides (mg/dL)	115.3 ± 96.5	140.1 ± 86.7	< 0.001
Total cholesterol (mg/dL)	171.1 ± 38.0	190.8 ± 37.1	< 0.001
HDL‐C (mg/dL)	47.7 ± 18.3	51.1 ± 14.0	< 0.001
LDL‐C (mg/dL)	101.1 ± 34.9	119.0 ± 34.0	< 0.001
Hs‐CRP	1.8 ± 7.2	1.4 ± 6.5	0.190
FPG (mg/dL)	128.5 ± 33.0	97.1 ± 9.9	< 0.001
HbA1c (%)	6.9 ± 1.1	5.6 ± 0.4	< 0.001
Insulin (μU/mL)	15.6 ± 19.5	11.3 ± 9.4	< 0.001
HOMA‐IR	5.1 ± 6.6	2.7 ± 2.3	< 0.001
≥ 2.5	532 (65.1)	741 (41.1)	< 0.001
Creatinine (mg/dL)	0.94 ± 0.71	0.85 ± 0.41	0.001
eGFR (mL/min/1.73 m^2^)	90.3 ± 28.9	95.5 ± 22.8	< 0.001
< 60	98 (10.9)	80 (4.1)	< 0.001
FIB‐4	1.46 ± 0.97	1.23 ± 1.35	< 0.001
> 1.3	421 (46.1)	652 (33.1)	< 0.001
> 2.67	72 (7.9)	80 (4.1)	< 0.001
CMRFs			
≤ 2 items	57 (6.2)	574 (29.2)	< 0.001
≥ 3 items	856 (93.8)	1395 (70.8)	

Abbreviations: ALT, alanine aminotransferase; AST, aspartate aminotransferase; BMI, body mass index; CMRFs, cardiometabolic risk factors; DBP, diastolic blood pressure; eGFR, estimated glomerular filtration rate; FIB‐4, fibrosis‐4 index; FPG, fasting plasma glucose; HbA1c, glycated hemoglobin; HDL‐C, high‐density lipoprotein cholesterol; HOMA‐IR, homeostatic model assessment for insulin resistance; Hs‐CRP, high‐sensitivity C‐reactive protein; IPTW, inverse probability of treatment weighting; LDL‐C, low‐density lipoprotein cholesterol; MASLD, metabolic dysfunction‐associated steatotic liver disease; SBP, systolic blood pressure; T2DM, type 2 diabetes mellitus.

The mean FIB‐4 score in T2DM patients was 1.46 ± 0.97, which was significantly higher than that in non‐T2DM patients (1.23 ± 1.35, *p* < 0.001), while the proportion of patients with advanced fibrosis (FIB‐4 > 2.67) was also significantly higher in the T2DM group (7.9% vs. 4.1%, *p* < 0.001).

### 3.3 Risk Factors for Advanced Fibrosis

**FIGURE 2:**
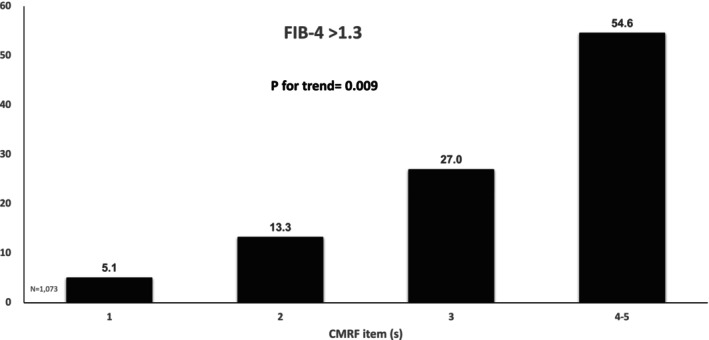
The proportions of significant fibrosis according to CMRF items.

The mean value of FIB‐4 was 1.31 ± 1.24. A total of 152 (5.3%) patients had advanced fibrosis, defined as FIB‐4 > 2.67. There was a significant linear trend between the presence of significant fibrosis and the items of CMRFs. The proportions of significant fibrosis were 5.1%, 27%, and 54.6% in patients with one CMRF, 3 CMRFs, and ≥ 4 CMRFs, respectively (*p* for trend = 0.009) (Figure 2). The subgroup analysis demonstrated that there's a significant linear trend between the CMRFs items and significant fibrosis in patients aged > 60 years, while the trend was not significant in patients aged ≤ 60 years.

Multivariate logistic regression analysis was then performed to identify the risk factors for prediction of advanced fibrosis, determining that the significant risk factors for advanced fibrosis in MASLD patients were age > 60 years (aOR = 12.08, 95% CI = 6.23–23.46, *p* < 0.001), elevated AST level (> 1 ULN, aOR = 1.13, 95% CI = 1.11–1.16, *p* < 0.001), elevated ALT level (> 1 ULN, aOR = 1.06, 95% CI = 1.05–1.09, *p* < 0.001), and T2DM (aOR = 1.51, 95% CI = 1.02–2.26, *p* = 0.04) (Table 3).

**TABLE 3:** Multivariate logistic regression analysis for FIB‐4 ≥ 2.67 in MASLD patients.

Variables	Crude OR (95% CI)	*p*	Adjusted OR (95% CI)	*p*
Age, ≥ 60 years	7.26 (4.67–11.30)	< 0.001	12.08 (6.23–23.46)	< 0.001
Male	1.15 (0.82–1.59)	0.420		
BMI ≥ 23 kg/m^2^	0.98 (0.58–1.64)	0.929		
AST (IU/L)	1.03 (1.02–1.03)	< 0.001	1.13 (1.11–1.16)	< 0.001
ALT (IU/L)	1.00 (1.00–1.01)	0.001	1.06 (1.05–1.09)	< 0.001
CMRFs				
Overweight/obesity	0.88 (0.51–1.52)	0.649		
Hypertension	1.42 (0.94–2.14)	0.093		
Hyper‐TG	1.02 (0.73–1.42)	0.911		
High HDL‐C	1.21 (0.86–1.69)	0.281		
Diabetes	1.90 (1.36–2.66)	< 0.001	1.51 (1.02–2.26)	0.041

Abbreviations: ALT, alanine aminotransferase; AST, aspartate aminotransferase; BMI, body mass index; CMRFs, cardiometabolic risk factors; FIB‐4, fibrosis‐4 index; HDL‐C, high‐density lipoprotein cholesterol; MASLD, metabolic dysfunction‐associated steatotic liver disease; TG, triglycerides.

### 3.4 The Biomarkers of CMRFs Predictive of Advanced Fibrosis Stage

**FIGURE 3:**
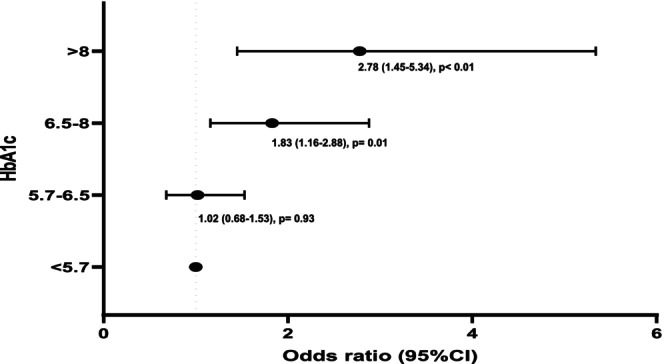
The adjusted odds ratios for advanced fibrosis prediction stratified by different HbA1c levels.

Among the various biomarkers, high‐sensitivity C‐reactive protein (hs‐CRP) and hemoglobin A1c (HbA1c) are closely associated with CMRFs and CVD prediction with extremely reliable and stable performance. Associations between these two biomarkers associated with disease severity were therefore investigated and evaluated as was the predictive role of HbA1c in advanced fibrosis in a dose‐dependent classification. The aORs for advanced fibrosis in patients of HbA1c 5.7%–6.5%, 6.5%–8.0%, and > 8.0% were 1.02 (95% CI = 0.68–1.53, *p* = 0.93), 1.83 (95% CI = 1.16–2.88, *p* = 0.01) and 2.78 (95% CI = 1.45–5.34, *p* = 0.002), respectively (Figure 3).

**FIGURE 4:**
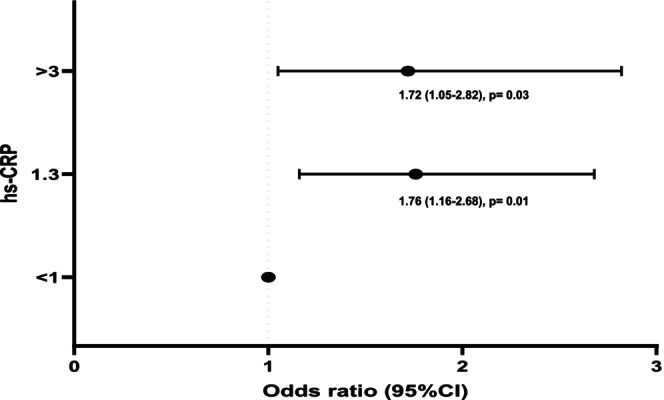
The adjusted odds ratios for advanced fibrosis prediction according to hs‐CRP levels.

Among the 2635 patients with available hs‐CRP data, 285 (10.8%) of them carried a high risk for CVD, defined as hs‐CRP > 3 mg/L. The proportion of patients carrying hs‐CRP > 3 mg/L was 15.3% (21/137) in the advanced fibrosis group, which was significantly higher than the FIB‐4 < 2.67 counterparts (10.6%, 264/2497, *p* = 0.04). Compared with 4.4% (85/1925) of advanced fibrosis in patients who had hs‐CRP < 1 mg/L, the proportions of advanced fibrosis in patients who had hs‐CRP 1–3 mg/L and > 3 mg/L were 7.5% (32/425, aOR = 1.76, 95% CI = 1.16–2.68, *p* = 0.01) and 7.4% (21/285, aOR = 1.72, 95% CI = 1.05–2.82, *p* = 0.03), respectively (Figure 4).

## 4 Discussion

This study investigated the epidemiological features of a MASLD‐based prospective cohort in a multicenter approach in Taiwan, demonstrating that nearly three‐fourths of the MASLD patients had glucose abnormalities. T2DM patients with MASLD carried a higher risk for concomitant CMRFs as well as advanced disease severity; moreover, T2DM patients had a higher risk for advanced fibrosis than their non‐T2DM counterparts. In addition, our results demonstrated that HbA1c level, the common biomarker of T2DM, significantly predicted advanced fibrosis in MASLD. This study is the first, to our knowledge, to demonstrate that HbA1c level could serve as a predictive biomarker for advanced fibrosis in MASLD. Of note, the predictive role of HbA1c became significant upon the HbA1c level becoming higher than 6.5%. As a result, in addressing the evidence showing the tight link between T2DM and the disease outcome of MASLD, this finding could pave the way for future implementation of a regional preventive strategy.

Liver fibrosis is the major determinant and the significant predictor of long‐term outcome in MASLD patients [15, 16]. A previous study demonstrated that MASH patients with advanced fibrosis had a significantly higher risk of all‐cause mortality as well as liver‐related mortality compared to MASH patients without fibrosis [17]. Among the risk factors for fibrosis progression, T2DM is a major one in MASLD patients while also increasing the risk of HCC development in the general population, even in patients without cirrhosis [18]. The cumulative burden of both common metabolic disorders correlates with an elevated risk of cirrhosis and HCC in a dose‐dependent manner [19, 20]. The current study echoed the findings showing that more than 30% of MASLD patients suffered from T2DM and three‐fourths of them were pre‐diabetic or T2DM cases. Meanwhile, T2DM patients exhibited a significantly worse disease severity, as evidenced by the higher mean FIB‐4 value and greater proportion of patients with advanced fibrosis compared to non‐T2DM patients. Resultantly, the presence of T2DM represents a significant risk factor in MASLD and should be assessed and managed with high priority while surveillance for advanced liver fibrosis should be prioritized in this patient group, especially for those who are elderly, have elevated ALT levels, or exhibit renal impairment. These findings are concordant with previous investigations that have emphasized the importance of liver enzyme levels, renal function, and age in liver fibrosis progression [19, 20]. Early identification of such high‐risk patients could facilitate timely interventions and help prevent the progression to cirrhosis and end‐stage liver disease [4].

T2DM per se is commonly a state of chronic inflammation and oxidative stress, both of which contribute to the activation of hepatic stellate cells and the progression of fibrosis [21]. The current study tested the commonly‐used cardiometabolic biomarkers for investigating their role in advanced fibrosis prediction. HbA1c has been regarded as the most reliable biomarker for T2DM, presenting its role from diagnosis, disease control status and outcome prediction. The UK Prospective Diabetes Study (UKPDS) found that a 1% reduction in mean HbA1c is associated with a 37% decrease in the risk of diabetes‐related microvascular complications [22]. Accordingly, this diabetes‐related biomarker could be applied for its potential in predicting the disease severity of MASLD since T2DM contributes mainly to disease progression in MASLD. Our results addressed the concept that HbA1c > 6.5% significantly predicted advanced fibrosis in MASLD patients. The aOD for advanced fibrosis reached 2.78 with HbA1c > 8.0%. These findings underscore the critical role of IR and metabolic dysfunction in the development and progression of MASLD, leading to more severe liver injury in patients with T2DM. Future long‐term follow‐up studies addressing the potential direct evidence between HbA1c and fibrogenesis are needed to clarify these interesting observations. Furthermore, the association between the biomarkers and cirrhosis also deserves investigation for early prediction of liver‐related outcomes.

ASCVD is the main cause of mortality in MASLD patients and disease screening is a must in clinical settings. Hs‐CRP is the surrogate biomarker for the activation of this pro‐inflammatory pathway mediated by IL‐1β and IL‐6., so is an acute‐phase biomarker for underlying systemic inflammation where an elevated hs‐CRP level is a prognostic marker for mortality and recurrence of ASCVD risk for primary and secondary prevention [12, 23]. Our study tested the correlation between high hs‐CRP level and disease severity with the results further addressing the close link between ASCVD risk and advanced fibrosis. Further study investigating the disease course, either progression or regression, dependent on the changes of hs‐CRP level in a longitudinal manner would be mandatory for this interesting issue.

MASLD is closely associated with and often precedes the development of CMRFs. The presence of CMRFs also confers a greater risk of progressive liver injury and major adverse liver outcomes [1, 24], as disease progression risk increases in the presence of CMRFs. Our recent study demonstrated that the presence of CMRFs significantly increased the risk of developing hypertension and T2DM in both SLD and non‐SLD groups, where MASLD was correlated with a 2.4‐ to 3.2‐fold higher odds of pre‐existing hypertension and T2DM, respectively [5].

Of note was that one‐fourth of MASLD patients carrying three or more items of CMRFs were of advanced fibrosis stage. The results were concordant with a previous large cohort study showing that the stepwise increase of CMRF items, beyond one CMRF item, was associated with disease severity [19]. Our previous community‐based study showed a significantly higher proportion of lean SLD patients with at least two CMRFs had a higher risk of advanced fibrosis than SLD/T2DM patients or SLD/obesity patients [2], and by extending these observations in comparing MASLD patients with normal and high BMI groups, it was determined that although the MASLD patients with normal BMI had lower prevalence of MetS and metabolic abnormalities than their high BMI counterparts, the significantly worse disease severity in the normal BMI group should be closely monitored and followed up. Our results also suggested the priority of periodic liver‐related‐event surveillance in the high‐risk MASLD patients by non‐invasive methods [25]. Further studies addressing the relevance between sonography‐based elastography and CMRFs should be performed for investigation of such non‐invasive methods in treatment application.

There were some limitations of the study. First, liver biopsy was not used as the standard for the diagnosis of hepatic steatosis and fibrosis stage; rather, noninvasive imaging and biomarker modalities were used to define fibrosis in the cohort. Of note, FIB‐4 has been widely adapted as a reliable non‐invasive biomarker for fibrosis stage assessment, which has been readily proposed by the guidelines of many societies. Second, our study was a cross‐sectional multicenter one without longitudinal validation and observation, so future studies investigating a long‐term cohort construction linked with liver‐ and non‐liver outcomes as well as the effect of cardiometabolic drugs are warranted in order to elucidate the unmet needs within this important global issue, while longitudinal research is also needed for further determination of these issues besides the current cross‐sectional results of the study.

In conclusion, the study demonstrated that a tight link exists between MASLD and T2DM where T2DM patients with MASLD carried a higher risk for concomitant CMRFs as well as worse disease severity. The cardiometabolic biomarkers such as HbA1c and hs‐CRP levels were significantly predictive of advanced fibrosis. Of note, the study demonstrated that HbA1c level could serve as a predictive biomarker for advanced fibrosis in MASLD, implying that diabetes control appears mandatory for patients with MASLD in a strategic precision‐preventive approach.

## Funding

The study was partly supported by Kaohsiung Medical University Hospital (KMUH‐DK(B)114004‐1, KMUH‐113‐3R09, KMUH‐114‐4M01) and Kaohsiung Medical University (KMU‐TB114003, KMU‐TC114A08). Ministry of Health and Welfare, Taiwan. (MOHW115‐TDU‐B‐222‐114011, 114‐2321‐B‐037‐006). Center of Excellence for Metabolic Associated Fatty Liver Disease, National Sun Yat‐sen University, Kaohsiung from The Featured Areas Research Center Program within the framework of the Higher Education Sprout Project by the Ministry of Education, Taiwan.

## Disclosure

All authors declare that they did not use generative AI in the manuscript preparation and review processes.

## Ethics Statement

Institutional Review Board of Kaohsiung Medical University Hospital approved the study.

## Conflicts of Interest

Jee‐Fu Huang is a consultant for Roche, Sysmex, Boehringer Ingelheim, Novo Nordisk, and Aligos. Speaker for Abbvie, Gilead, Merck, Sysmex, Boehringer Ingelheim, and Novo Nordisk. Chung‐Feng Huang is a speaker for Abbvie, BMS, Bayer, Gilead, Merck, and Roche. Chia‐Yen Dai is a consultant of Abbvie and Roche. Speaker for Abbvie, Gilead, and Roche. Ming‐Lung Yu is a research grant from Abbott, BMS, Merck, and Gilead; Consultant of Abbvie, Abbott, Ascletis, BMS, Merck, Gilead, and Roche; Speaker for Abbvie, Abbott, BMS, Merck, Gilead, and IPSEN. Wan‐Long Chuang is a consultant of Gilead, AbbVie, BMS, and PharmaEssentia; speaker for Gilead, AbbVie, BMS, and PharmaEssentia. The other authors declare no conflicts of interest.

## Data Availability

The data that support the findings of this study are available on request from the corresponding author. The data are not publicly available due to privacy or ethical restrictions.
